# Intraoperative Findings of Elective Laparoscopic Cholecystectomy in Diabetics Versus Nondiabetics: A Comparative Study

**DOI:** 10.7759/cureus.20886

**Published:** 2022-01-03

**Authors:** Ashish Luthra, Aparna Behura, Chinmaya R Behera, Amaresh Mishra, Subrat Mohanty, Bandita Panda

**Affiliations:** 1 Department of Surgery, Nemi Chand Medical College and Hospital, Panipat, IND; 2 Department of Pathology, Kalinga Institute of Medical Sciences, Bhubaneswar, IND; 3 Department of Surgery, Kalinga Institute of Medical Sciences, Bhubaneswar, IND; 4 Department of Surgery (Pediatric Surgery), Kalinga Institute of Medical Sciences, Bhubaneswar, IND; 5 Department of Research and Development, Kalinga Institute of Medical Sciences, Bhubaneswar, IND

**Keywords:** diabetes, intraoperative finding, cholecystitis, complications, gallstone disease

## Abstract

Objectives

Diabetes mellitus predisposes to the formation of gallstones. Surgery for gallstone disease (GSD) in diabetic patients also carries more risk compared with nondiabetic patients. The objective of the present study was to evaluate the intraoperative findings of elective laparoscopic cholecystectomy in diabetics and nondiabetics.

Methods

This study was carried out for two years in the department of general surgery as a prospective observational study. Two groups of patients with uncomplicated gallstone disease were recruited: one group included 75 diabetics and the other one included 75 nondiabetics. The two study groups were matched by age and gender. Detailed history and intraoperative findings and their outcomes were recorded. Patients with emergencylaparoscopiccholecystectomy for acute cholecystitis and its complications and cholecystectomy associated with common bile duct (CBD) stones were excluded from the study.

Results

The results of elective laparoscopic cholecystectomy in the groups were compared. No demographic differences were found between the groups. Adverse intraoperative findings such as thick-walled gallbladder (GB), pericholecystic collections, and adhesions to the surrounding structures, surgical difficulties, modification to subtotal cholecystectomy, and open conversion were more frequent in diabetic patients than in nondiabetic patients.

Conclusion

Laparoscopic cholecystectomy in diabetic patients had more adverse intraoperative findings in comparison to nondiabetic patients. However, in elective laparoscopic cholecystectomy, good preoperative preparation and meticulous surgical technique are mandatory to achieve similar outcomes between the groups.

## Introduction

Laparoscopic cholecystectomy is the gold standard for all symptomatic gallstone disease (GSD), and its prevalence is higher in patients with diabetes compared with those without. Diabetics are more susceptible to infections, as high blood sugar levels can weaken the patient’s immune system defense and hamper normal healing mechanisms.

Diabetics along with various factors such as obesity-associated diabetes, insulin resistance, high triglyceride level, and autonomic neuropathy interplay in the formation of gallstones [1]. Diabetes is fast increasing globally, with a great concern for India due to its large population with changing lifestyle and dietary habits. The morbidity and mortality in diabetes are mostly due to severe inflammation in diabetes and also silent presentation. Therefore, diabetes is considered an independent risk factor for intraoperative complications, but in well-controlled diabetes with good preoperative preparation and surgical technique, the outcome is the same in both diabetic and nondiabetic groups [2,3].

Because of the increased incidence of gallstones and the use of laparoscopic cholecystectomy, there is a great need to study the adverse intraoperative findings and surgical outcomes in diabetics versus nondiabetics with respect to its safety and efficacy.

This study aims to compare various intraoperative findings of laparoscopic cholecystectomy in diabetics and nondiabetics and its overall surgical outcome.

## Materials and methods

This prospective observational study was carried out in the Department of General Surgery, Kalinga Institute of Medical Science in Bhubaneswar, a tertiary care hospital in Eastern India, from August 2017 to July 2019.

Inclusion criteria

The study included all patients undergoing elective laparoscopic cholecystectomy during the study period. The total numbers of patients included in the study were 150 (75 each in both diabetic and nondiabetic groups with comparable demographic profiles).

Exclusion criteria

Patients undergoing emergencylaparoscopiccholecystectomy for acute cholecystitis and its complications and cholecystectomy associated with common bile duct (CBD) stones were excluded.

For symptomatic diabetic patients, preoperative glucose such as fasting blood sugar, two-hour postprandial blood sugar level, and HbA1c were measured. The type of diabetes (type 1 or type 2), status of diabetes, complications, and duration of their disease on admission were recorded. Patients with oral hypoglycemic drugs or insulin were also recorded. Disease-specific routine preoperative workups, such as liver function tests and ultrasound, to know the status of the gallbladder (GB), were conducted. MRCP was done selectively in patients if there was any suspicion of CBD stone and in patients with deranged liver function tests. Other tests, such as routine blood tests, chest X-rays, and ECG, were done in all patients. Cardiac echocardiogram study and pulmonary function test and other tests were done selectively depending on specific circumstances.

Operative findings such as difficult anatomy, complications, duration of surgery, and conversion to open of all patients in both groups were recorded at the time of surgery. Operative events such as injury to the common bile duct (CBD) or bowel, uncontrolled bleeding, and any perforation of the gallbladder with bile leak into the abdominal cavity were recorded.

The following intraoperative findings of both groups that were taken into consideration were recorded: status of the gallbladder (distended or contracted gallbladder), wall thickness, pericholecystic adhesions, Calot’s anatomy, impaction of stone in the neck, duration of surgery, bleedings, and bile duct and other organ injuries. Surgical outcomes with modifications of surgery such as subtotal cholecystectomy, open conversion, drain placement, and recovery from anesthesia were noted.

All data were analyzed using statistical analysis such as mean, standard deviation, and p-value (p-value < 0.05 was considered to be significant) for data validation. All analyses were done with the help of the standard statistical software STATA version 13.1.

## Results

A total of 150 samples, including the diabetic and nondiabetic groups (75 patients in each group), were included in the study; the diabetic group patients were in the age group of 18-75 years, with a mean age at the time of surgery of 41.4 ± 8.53 years, and the nondiabetic group patients were in the age group of 22-75 years, with a mean age at the time of surgery of 40.24 ± 10.34 years. Of the 75 patients in the diabetic group, there were more male patients (77.3%) than female patients (22.6%). In the nondiabetic group, of the 75 patients, there were more female patients (58.6%) than male patients (41.3%). Both groups had a comparable body mass index (BMI) and distribution of various comorbidities in the study population. The American Society of Anesthesiologists (ASA) fitness category did not significantly differ between the two groups.

**Figure 1:**
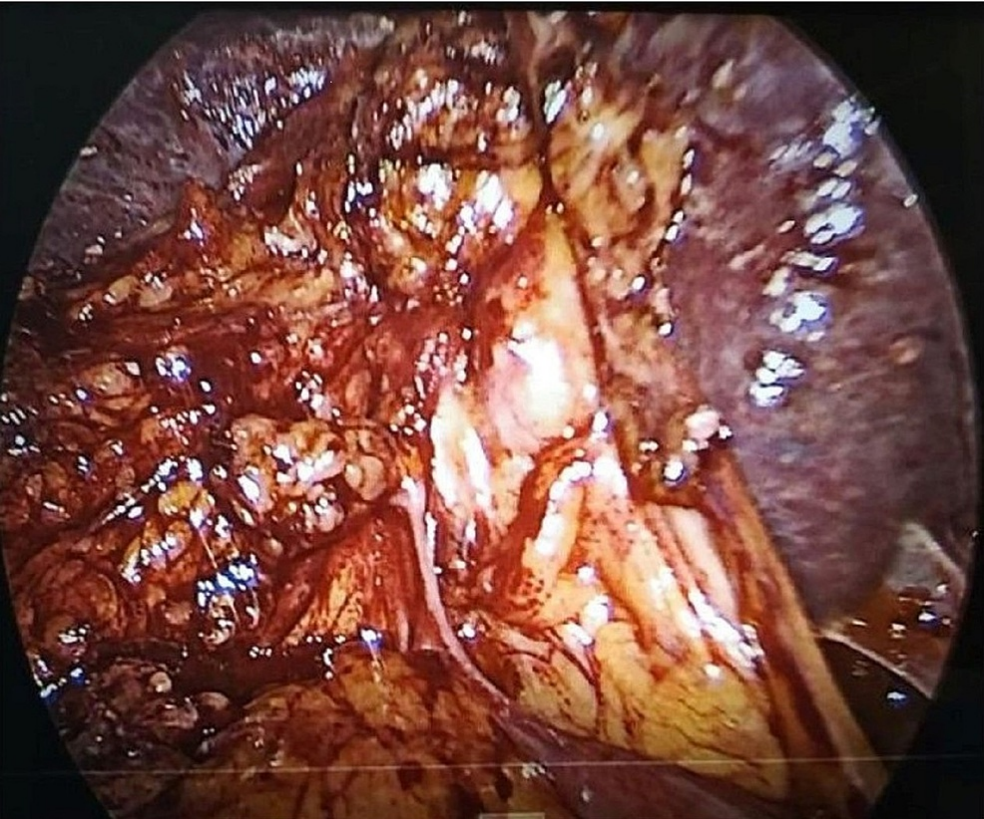
Pericholecystic adhesion in a diabetic patient

**Figure 2:**
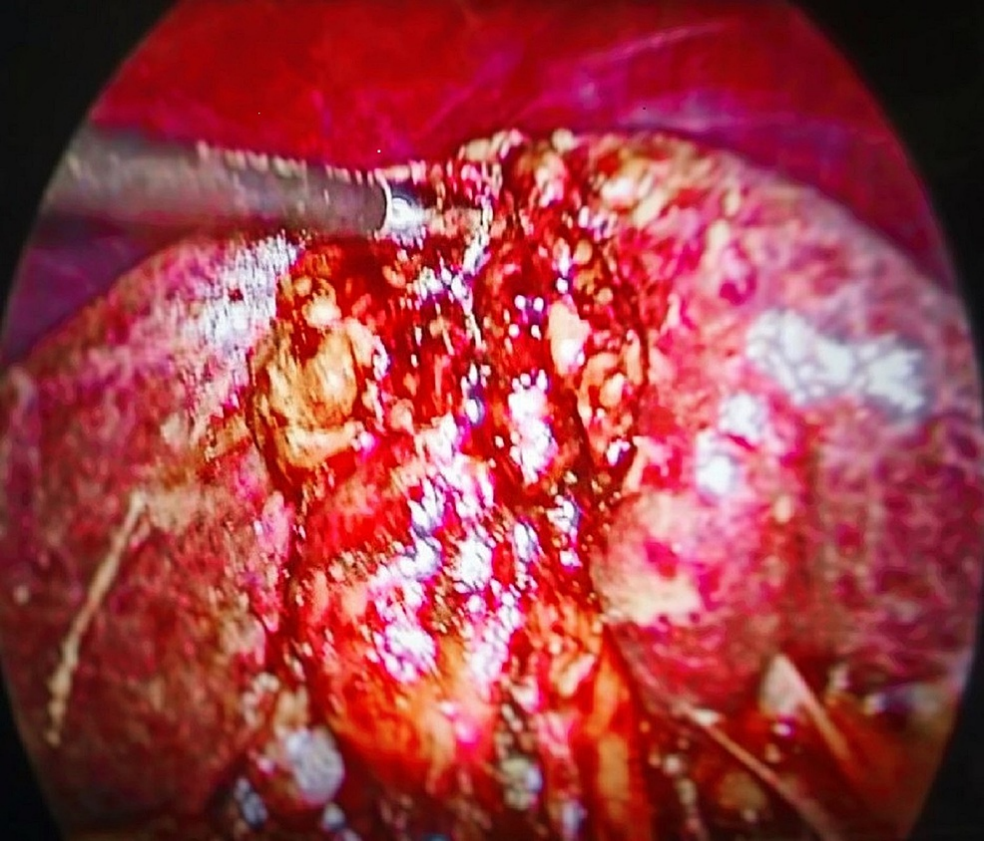
Contracted gallbladder in a diabetic patient

Pericholecystic adhesions (Figure 1), contracted gallbladder (Figure 2), gallbladder wall thickening, and frozen Calot’s triangle were observed significantly more among diabetic patients in comparison with nondiabetic patients. However, impaction of stone in Hartman’s pouch was present in six patients (8%) in the diabetic group in comparison with four patients (5.3%) in the nondiabetic group (Table 1).

**Table 1:** Comparative intraoperative parameters between the two groups (diabetic and nondiabetic groups)

Intraoperative parameters	Diabetic (%) (N = 75)	Nondiabetic (%) (N = 75)	Significance (p-value < 0.05)
Pericholecystic adhesion	56	46.6	0.00059
Contracted GB	38.60	17.30	0.00001
Thickened-walled GB	33.30	21.30	0.000207
Calot’s triangle frozen	13.30	13.30	(NS)
Impaction of stone in Hartman’s pouch	8	5.33	0.3632
Subtotal cholecystectomy	5.33	2.66	0.3910
Conversion to open	4	1.33	0.42265
Duration of surgery (more than 90 minutes)	69.30	36	0.00001
Drain	18.60	12	0.0905
Recovery from anesthesia (more than 20 minutes)	60	24	0.0001

**Figure 3:**
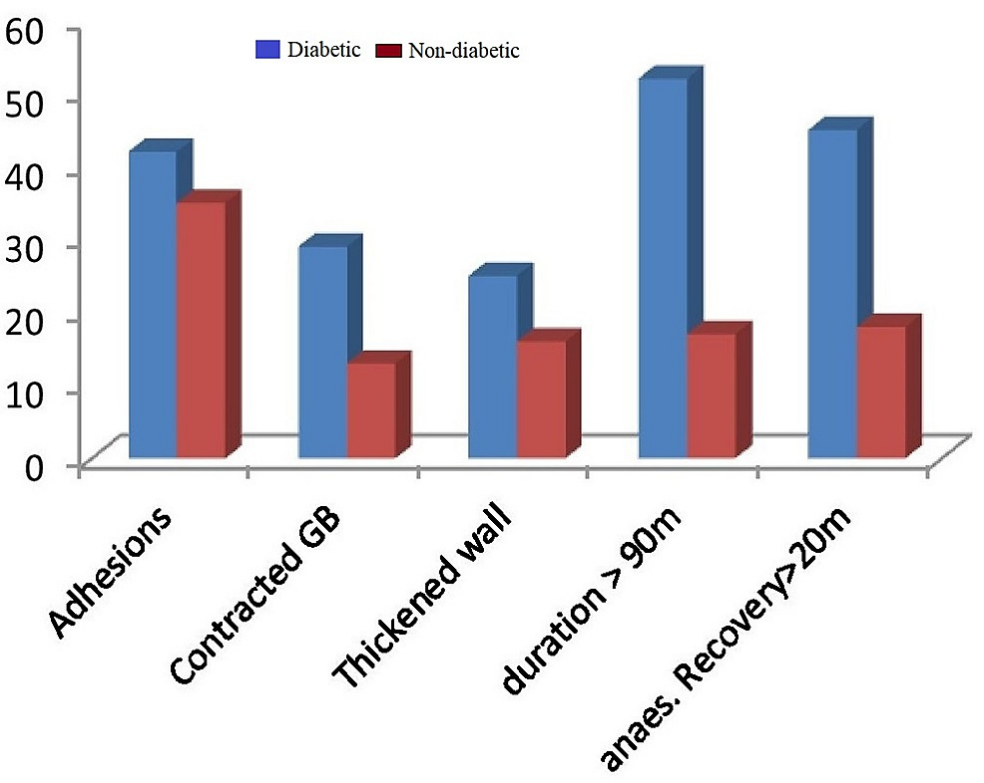
Comparative intraoperative findings between the diabetic and nondiabetic groups

Subtotal cholecystectomy was done in four patients (5.3%) in the diabetic group and two patients (2.6%) in the nondiabetic group. The reason for subtotal cholecystectomy was frozen Calot’s anatomy in both groups of patients. Conversion to open procedure was done in 4% of diabetic patients due to dense adhesions, altered anatomy, and inability to proceed to laparoscopy. The mean duration of surgery was taken as 90 minutes, and it exceeded in 52 diabetic patients (69.3%) and 27 nondiabetic patients (36%). A drain was given in 14 patients (18.6%) in the diabetic group and nine patients (12%) in the nondiabetic group. A tube drain was inserted in all patients with subtotal cholecystectomy and open conversion. Recovery from anesthesia was more than 20 minutes in 45 patients (60%) in the diabetic group and 18 patients (24%) in the nondiabetic, which was statistically significant. The reason for delayed anesthesia recovery was various intraoperative and metabolic factors in diabetes. Intraoperative injury was not reported in CBD or surrounding organs (Figure 3).

## Discussion

Gallbladder disease is a worldwide concern. In India, the prevalence of gallstone disease is around 10%-20%, more in females and progressively rising over the years [4]. This may be linked to changes in dietary habits and also increased detection due to more widespread ultrasound evaluation of abdominal complaints.

In a large population-based study in Taiwan, Liu et al. found that the incidence of gallstone disease (GSD) and subsequent cholecystectomy was 18.65% and 17.15% in the diabetic and control group, respectively [5]. Male sex and increasing age also contribute to increased incidence, irrespective of the diabetes status [5]. The relationship between diabetes and gallstone disease (GSD) is due to increased gallbladder volume that predisposes to bile stasis and stone formation [6]. Hyperlipidemia contributes to gallstone formation and is more observed in diabetics [7]. Increased cholesterol index and reduced gallbladder motility also contribute to stone formation in diabetes [8].

Laparoscopic cholecystectomy has become the gold standard treatment for gallstone disease [9]. It has less pain, reduced length of hospital stay, and early return to work and is cosmetically more acceptable. Increasing age and male sex are associated with more adverse intraoperative findings, adding to the surgical difficulty. The possibility of multiple attacks of cholecystitis is more with age, and Vivek et al. found that patients more than 65 years old were having more adhesions with difficult surgery [10]. In a gender-specific study by Ambe et al., male sex was a significant predictor of inflammation and difficult surgery [11]. However, in our study, the results were similar in both males and females across both groups. Similar difficulty with a bodyweight of more than 65 kg and BMI > 30 kg/m^2^ was observed [10,12]. In diabetes, more intraoperative difficulties were observed for those who had HbA1c of more than 6 [12].

The duration of surgery of diabetic patients was significantly higher than nondiabetic patients because of more adverse intraoperative findings. Intraoperative findings such as pericholecystic adhesions, contracted gallbladder, thickened wall, impacted stone in Hartman’s pouch, and frozen Calot’s anatomy are more often seen in diabetics. All these factors contribute to operative difficulty, increased time, complications, and also open conversion. This was reflected in our study populations, except for frozen Calot’s anatomy, similar in both groups. Different pain sensations and less sensitivity in diabetic neuropathy also result in a delayed diagnosis [13]. This leads to repeated silent attacks of cholecystitis.

The intraoperative decisions for modification were subtotal cholecystectomy and open conversion. Subtotal cholecystectomy was done in obscured Calot’s triangle anatomy, where a critical view of safety could not be achieved and it is a better alternative. In a meta-analysis, Elshaer et al.’s results were in favor of laparoscopic subtotal cholecystectomy [14]. The only problem was more bile leak and a possibility of stone formation of retained Hartman’s pouch. In our small series of a total of six patients, four patients were diabetic and the remaining two patients were nondiabetic, suggesting the more operative difficulty in diabetes. The possibility of stone formation is dependent on the residual infundibulum, more with 3-4 cm, as it forms the pouch and can be minimized if the distance is maintained within 1 cm [15]. Fenestration is an alternative method of ligating the cystic duct from inside the gallbladder, although less often preferred. Open conversion is considered as the last option when the gallbladder cannot be grasped or retracted in a buried gallbladder compromising all safety measures of cholecystectomy.

In 2010, Paajanen et al., from Finland, suggested that the comorbidities of diabetes, especially renal disease, were associated with a higher risk of complications. In their observations, 16% of their diabetic patients required conversion to open, compared with only 7% of their nondiabetic controls (p-value < 0.001) [16]. In our study, 4% of the diabetic patients were converted to open compared with nondiabetic patients (1.3%). The results were comparable and not statistically significant (p-value = 0.42265). The factors associated with conversion were dense adhesion with difficulty in grasping, uncontrolled bleeding, and unsatisfactory progress of laparoscopy. The open conversion is variable and depends on many factors [17]. Routine drain placement is controversial; however, a drain was selectively placed in cases suspected of bile leak or bleeding. In this study, a drain was placed in all subtotal cholecystectomy and open conversion and selectively in difficult cholecystectomy in anticipation of possible bile leak or bleeding. A total of 14 diabetic patients and nine nondiabetic patients had drain placement, suggesting more operative difficulty in diabetes.

Common bile duct injury is the most feared complication of laparoscopic cholecystectomy, and fortunately, the present study had no such injury. Adequate anatomical knowledge of biliary tact with surgical experience and judgment are essential prerequisites in avoiding CBD injury. Recovery from anesthesia was prolonged more than 20 minutes in 45 patients with diabetes and 18 patients without diabetes. This increased duration in diabetes is due to a various interplay of metabolic factors and pharmacokinetics in drugs with glycemic control [18].

In 2006, a study conducted by Ibrahim et al. in Singapore found that poorly controlled diabetes (elevated HbA1c > 6) was with increased risk for converting to open procedure (p-value < 0.038) [12]. They thought that poorly controlled blood sugar levels lead to severe inflammation and hence severe adhesions, distorting the anatomy in patients. Also, patients who had conversion were found to have significantly higher rates of complications postoperatively.

Walsh et al. reported no significant increase in operative morbidity in diabetics when compared to the general population [19]. They thought that diabetics have increased morbidity primarily because they are older and have other medical problems as well. However, careful preoperative preparation, meticulous intraoperative surgical technique, and cautious postoperative care are mandatory to achieve this outcome.

The number of patients enrolled is not adequate to substantiate a definite conclusion, which is the major limitation of this study. The observations were made only in elective laparoscopic cholecystectomy cases, and emergency patients were excluded from this study. Since diabetics are more prone to complications such as perforated GB/gangrenous GB in the acute stage, further studies are needed to establish a true picture of the efficacy and safety of laparoscopic cholecystectomy in diabetics.

## Conclusions

Elective laparoscopic cholecystectomy in diabetics had more adverse intraoperative findings, such as thickened gallbladder, pericholecystic adhesions, and contracted gallbladder, than in nondiabetics. Also, conversion to open and time taken for recovery of anesthesia was observed to be more in the diabetic group. However, in well-controlled diabetes, elective surgery does not appear to have a bad prognosis as compared to age and sex-matched controlled groups found in this study. Careful preoperative preparation and meticulous intraoperative surgical techniques with experience and judgment are mandatory to achieve a good outcome. As emergency laparoscopic cholecystectomy for acute and complicated cholecystitis had not been included in this study, the true picture of surgical outcome is inadequately reflected. The major limitation of the study is that the number of patients enrolled was not adequate, and the male gender is seen to have more difficulties than the female one while undergoing the said procedure; therefore, more studies with a large population are required to substantiate a definite conclusion.
